# Influences of nitrogen, phosphorus and silicon addition on plant productivity and species richness in an alpine meadow

**DOI:** 10.1093/aobpla/plv125

**Published:** 2015-11-14

**Authors:** Danghui Xu, Xiangwen Fang, Renyi Zhang, Tianpeng Gao, Haiyan Bu, Guozhen Du

**Affiliations:** 1 State Key Laboratory of Grassland Agro-ecosystems/School of Life Science, Lanzhou University, No. 222, South Tianshui Road, Lanzhou, Gansu 730000, China; 2 Centre of Urban Ecology and Environmental Biotechnology, Lanzhou City University, Lanzhou 730070, China

**Keywords:** Aboveground primary productivity (APP), community composition, nitrogen enrichment, plant functional group, silicon nutrient, species richness

## Abstract

Fertilization, especially with nitrogen (N), increases aboveground primary productivity (APP), but reduces plant species richness at some level. Silicon (Si) fertilization alone, or with addition of N or phosphorus (P), has multiple direct and indirect beneficial effects on plant growth and development, both for individuals and the whole community. This study aimed to examine the effects of Si, N, P, NSi and PSi combinations on APP and species richness of the community and of four functional groups in an alpine meadow. The results showed that plots fertilized with Si in combination with either N or P had higher APP than when fertilized with N or P alone. Addition of N or P increased APP, and the higher APP occurred when the highest level of N was added, indicating co-limitation of N and P, with N being most limiting. Silicon fertilization alone or with addition of N increased the APP of grasses and forbs. Nitrogen addition decreased the community species richness; Si with addition of N alleviated the loss of species richness of the whole community and the forbs group. For the four functional groups, N or P addition increased the species richness of grasses and decreased that of forbs. Our findings highlight the importance of Si in improving APP and alleviating N fertilization-induced biodiversity loss in grasslands, and will help improve our ability to predict community composition and biomass dynamics in alpine meadow ecosystems subject to changing nutrient availability.

## Abstract (precis)

Plots in an alpine meadow fertilized with Si in combination with either N or P had higher aboveground primary productivity (APP) and higher species richness than when fertilized with N or P alone. Our finding highlights the importance of Si in improving APP and alleviating N fertilization-induced biodiversity loss in grasslands, and will help improve our ability to predict community composition and biomass dynamics in alpine meadow ecosystems subject to changing nutrient availability.

## Introduction

Climatic conditions such as temperature and moisture limit plant productivity and nutrient cycling in cold regions, especially in alpine meadow, which leads to a strong nitrogen (N)- and phosphorus (P)-limitation in plant growth (Tamm 1991; Nadelhoffer *et al*. 1992). Plant communities are especially sensitive to N or P addition, and changes in the plant community structure are often mediated through increased productivity following addition of N or P (Clark *et al*. 2007). On the other hand, species-specific response to increasing N or P (Pennings *et al*. 2005) may lead to changes in plant community structure rather than an increase in productivity (Bret-Harte *et al*. 2008).

Considerable evidence indicates that change in N and P availability can dramatically alter local species composition, plant community structure and biological diversity (Gough *et al*. 1994; Stevens *et al*. 2004). Biological diversity plays an important role in the functioning and sustainability of ecosystems (Tilman *et al*. 1996). Given that anthropogenic activities have greatly increased N availability globally (Vitousek *et al*. 1997), it is essential to find an element that will not only increase aboveground primary productivity (APP) but will also slow down the loss of biological diversity.

Silicon (Si) is the second most abundant element in the soil. It is present in plants in amounts equivalent to those of such macronutrient elements as calcium, magnesium and P, and in grasses often at higher levels than any other inorganic constituent (Epstein 1999). Silicon fertilization is widely used to enhance crop production and improve crop quality (Epstein 1999; Liang *et al*. 2006). This effect of Si has been traditionally attributed to an increase in the erectness of leaves, allowing better light transmittance through plant canopies and thus indirectly improving whole-plant photosynthesis (Tamai and Ma 2008) and N use efficiency (Detmann *et al*. 2012).

In general, addition of Si has been recommended for improving the resistance of plants to abiotic and biotic stresses (Ma 2004; Ma and Yamaji 2006; Pavlovic *et al*. 2013). For example, Si deposition in the cell walls of root endodermal cells may contribute to the maintenance of an effective apoplastic barrier and thereby improve plant resistance to disease and drought stresses (Lux *et al*. 2003; Hattori *et al*. 2005), while intra- and extracellular deposition of aluminosilicates in roots and shoots is thought to protect some species from potential aluminium toxicity (Hodson and Evans 1995; Jansen *et al*. 2003; Wang *et al*. 2004). However, to our knowledge, the possible influence of Si on plant community composition and productivity in alpine meadow has not been tested so far. In this study, we test the hypothesis that there are high differences in species richness and APP of the community and four functional groups in an alpine meadow under different (N, P, Si, NSi and PSi fertilization) fertilization, and Si with N or P added has a more beneficial effect on species richness and APP than fertilization with N or P alone. To test this hypothesis, our objectives were (i) to evaluate the effects of N, P and Si addition alone and interactive effects of NSi and PSi on species richness and APP of the community, (ii) to identify whether there were any different responses of species richness and APP of the four functional groups in different treatments and (iii) to elucidate whether there existed beneficial interaction effects of NSi or PSi on species richness and APP of the community and different functional groups.

## Methods

### Site description

The study was conducted in the Research Station of Alpine Meadow and Wetland Ecosystems of Lanzhou University, which is located in Maqu, on the eastern Tibetan Plateau of China (33°58′N, 101°53′E; 3500 m above sea level). This area has been fenced and protected since 1999. Mean annual temperature is 1.2 °C (ranging from minima of −10 °C in January to 11.7 °C in July and growing season maxima of 23.6–28.9 °C). The average yearly precipitation is 620 mm (35-year average from the Maqu Weather Station), 2580 h of solar radiation annually and 100 days of frost-free period in a year (Chu *et al*. 2008). The plant community represents a typical and diverse alpine meadow with an alpine meadow soil, dominated by grasses such as *Festuca ovina*, *Poa poophagorum* and *Elymus nutans*; sedges such as *Scirpus pumilus* and *Kobresia capillifolia*; forbs such as *Anemone rivularis*, *Trollius farreri* and *A. obtusiloba* and legumes such as *Astragalus polycladus* and *Gueldenstaedtia verna* (Li *et al*. 2011).

### Experimental treatments

The sample sites were in an open, flat area where there was little slope. Livestock was entirely excluded from all of the sites during the growing season from April to October to avoid grazing; however, some low-level grazing was allowed during the hay-stage in the winter from 2000 onwards. The experiment was laid out in a completely randomized block design in early May 2012 and 2013. Sixteen treatments consisted of single nutrient additions of Si, N and P at three levels, and additions of Si and N or P at three levels (Table 1). The amount and level of N addition were according to Luo *et al*. (2006) and P was, according to Yang *et al*. (2013), what they had done in alpine meadow. Six randomly selected plots (5 × 5 m^2^) were established in each treatment, resulting in 96 plots. All plots were separated by 2 m of buffer zones without any fertilization. The plots were randomly arranged in every block.

**Table 1.** Addition levels and amounts of Si, N and P.

Different treatments	Addition levels of N, P and Si (g m^−2^)			Addition amounts of N, P and Si (g m^−2^)		
	Ammonium nitrate	Calcium superphosphate	Silicic acid	N	P	Si
CK	0	0	0	0	0	0
Si1	0	0	2	0	0	0.718
Si2	0	0	4	0	0	1.436
Si3	0	0	6	0	0	2.154
N1	20	0	0	7	0	0
N2	40	0	0	14	0	0
N3	60	0	0	21	0	0
N1Si2	20	0	4	7	0	1.436
N2Si2	40	0	4	14	0	1.436
N3Si2	60	0	4	21	0	1.436
P1	0	40	0	0	4.92	0
P2	0	80	0	0	9.84	0
P3	0	120	0	0	14.76	0
P1Si2	0	40	4	0	4.92	1.436
P2Si2	0	80	4	0	9.84	1.436
P3Si2	0	120	4	0	14.76	1.436

Samples were taken annually in early September of 2012 and 2013, when biomass had reached its highest, from one 0.5 × 0.5 m quadrat from every plot. The quadrat location was randomly selected with the constraint that it was at least 0.5 m from the edge to avoid marginal effect. Every ramet was counted for each species, all plants were clipped at the soil surface and separated into the four functional groups—grasses, sedges, forbs and legumes—and put in marked paper bags per quadrat. The dry biomass of every functional group in every quadrat was weighed after being oven-dried at 80 °C for 48 h to constant weight. Counting individuals of clonal plants is very difficult, so we counted apparent clusters of stems as an individual (a ramet in most cases). Clones of temperate, caespitose grasses were organized as assemblages of autonomous ramet hierarchies, rather than as a sequence of completely integrated ramets. The benefits of physiological integration were restricted to individual ramet hierarchies, which consist of approximately three connected ramet generations. The summed biomass of the four functional groups was used as an estimate of community APP (Ren *et al*. 2010).

### Statistical analysis

All statistical analyses were performed using SPSS 13.0 for windows (SPSS Inc., Chicago, IL, USA). Before analysis, all data were tested for normality and all data met the normality distribution. The effects of the fertilization treatment on APP of the community and four functional groups, and species richness of the community and four functional groups in 2012 and 2013 were tested, respectively, by one-way analysis of variance (ANOVA) with least significant differences (LSD test) at *P* < 0.05. Two-way ANOVA was used to determine the interaction effects that are caused by N or P alone and by N or P in combination with Si in species richness of the community and four functional groups, and APP of the community and four functional groups. Two-way ANOVAs were also performed to determine the effects of fertilization (control, three levels of Si, three levels of N, three levels of N, three levels of NSi and three levels of PSi), year (2012 and 2013) and their interaction on the following response variables: APP and species richness of the community and the four functional groups. If main effects or interactions were significant, we then preceded with multiple comparison tests to compare differences among means using least significant differences (LSD test) at *P* < 0.05.

## Results

### Aboveground primary productivity of community

**Figure 1.**
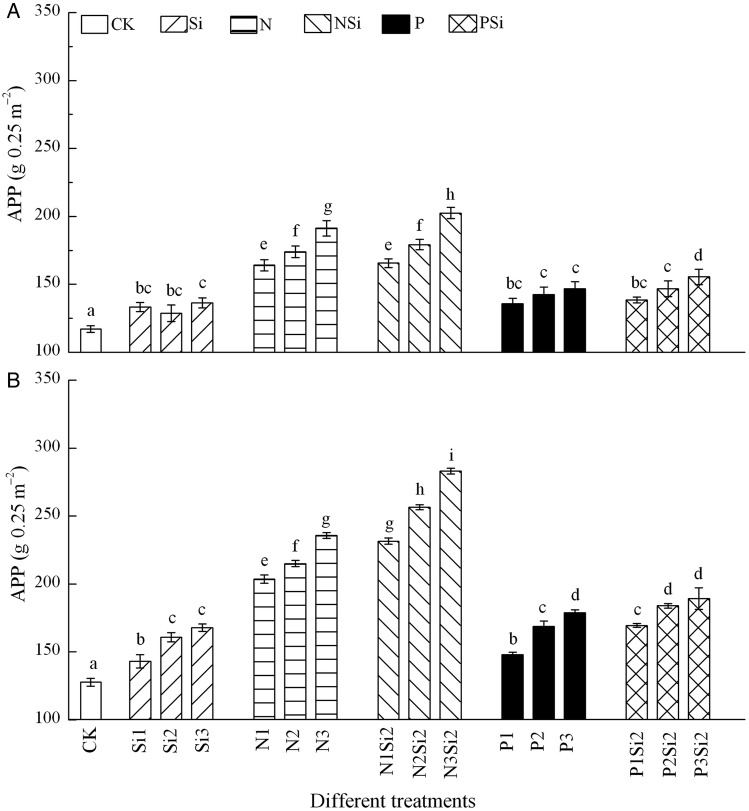
Mean (±1 SE) APP (g 0.25 m^−2^) of the community after Si, N and P addition in 2012 (A) and 2013 (B), *n* = 6. Different letters above bars indicate significant difference between different treatments.

Aboveground primary productivity increased with the increasing levels and amounts of Si (*F* = 11.524, df = 2, *P* = 0.001), N (*F* = 38.803, df = 2, *P* < 0.001) and P (*F* = 16.138, df = 2, *P* < 0.001) addition, both in 2012 (Fig. 1A) and 2013 (Fig. 1B). Addition of Si combined with N (*F* = 154.539, df = 5, *P* < 0.001) or with P (*F* = 23.6, df = 5, *P*< 0.001) produced a stronger response than N or P addition alone. Two-way ANOVA demonstrated significant interaction effects between N and Si in 2012 (*F* = 165.1, df = 1, *P* = 0.041) and 2013 (*F* = 197.3, df = 1, *P* = 0.028) (Fig. 1), and between 2012 and 2013 in different treatments (Table 2).

**Table 2.** Degrees of freedom (df), *F* values and probabilities of two-way ANOVA between 2012 and 2013.

Character	Source of variation	Treatment (T)	Year (Y)	T × Y
	df	15	1	15
APP				
Community	*F* value	398.28	2153.27	41.50
	Probability	<0.001	<0.001	<0.001
Grasses	*F* value	564.70	1068.44	51.22
	Probability	<0.001	<0.001	<0.001
Sedges	*F* value	16.55	376.52	11.36
	Probability	<0.001	<0.001	<0.001
Legume	*F* value	178.96	57.87	11.90
	Probability	<0.001	<0.001	<0.001
Forbs	*F* value	24.62	24.30	21.45
	Probability	<0.001	<0.001	<0.001
Species richness				
Community	*F* value	26.20	36.30	4.83
	Probability	<0.001	<0.001	<0.001
Grasses	*F* value	17.22	0.126	1.59
	Probability	<0.001	0.732	0.082
Sedges	*F* value	1.157	11.81	1.39
	Probability	0.311	<0.001	0.154
Legume	*F* value	6.413	0.317	4.042
	Probability	<0.001	0.574	<0.001
Forbs	*F* value	46.519	38.890	5.848
	Probability	<0.001	<0.001	<0.001

### Species richness of community

**Figure 2.**
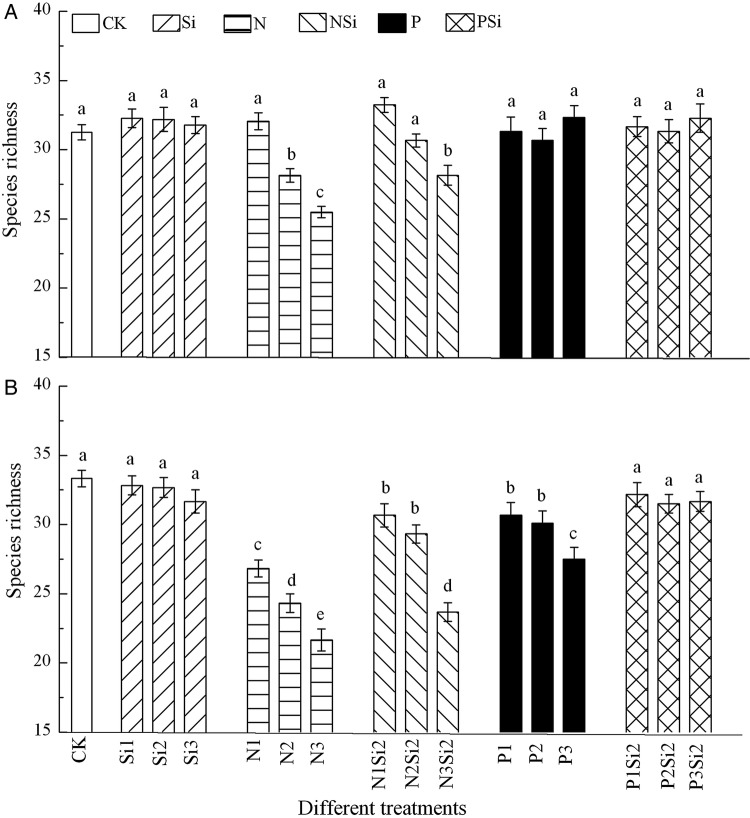
Mean (+1 SE) species richness of the community after Si, N and P addition in 2012 (A) and 2013 (B), *n* = 6. Different letters above bars indicate significant difference between different treatments.

Silicon addition alone and in combination with P did not affect the species richness of the community in either 2012 (*F* = 1.037, df = 6, *P* = 0.475) (Fig. 2A) or 2013 (*F* = 1.754, df = 6, *P* = 0.192) (Fig. 2B). Silicon in combination with N (*F* = 19.547, df = 5, *P*< 0.001) or P (*F* = 3.788, df = 5, *P* = 0.009) resulted in higher species richness than N or P addition alone in 2013. A highly significant interaction between Si and N was found in 2012 (*F* = 24, df = 1, *P* = 0.046) and 2013 (*F* = 47, df = 1, *P* = 0.021) (Fig. 2A).

### Aboveground primary productivity of four functional groups

**Figure 3.**
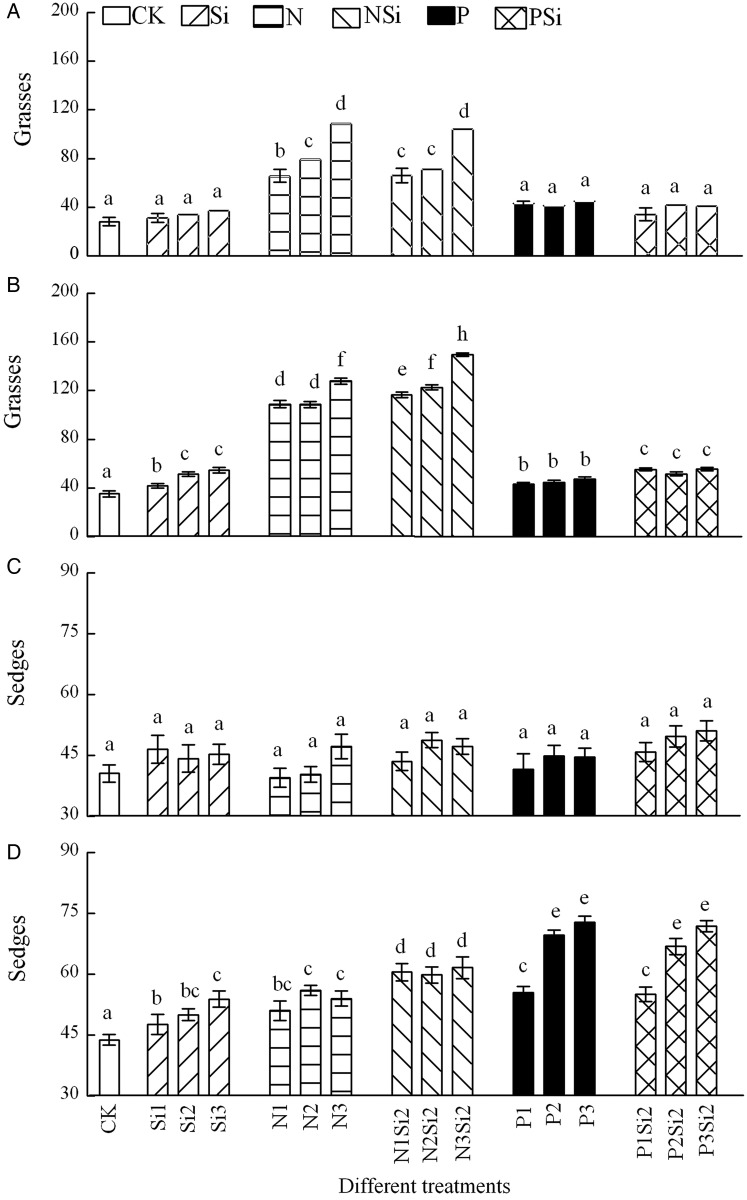
Aboveground primary productivity in groups of grasses (A and B) and sedges (C and D) after Si, N and P addition in 2012 (A and C) and 2013 (B and D), *n* = 6. Different letters above bars indicate significant difference between different treatments.

**Figure 4.**
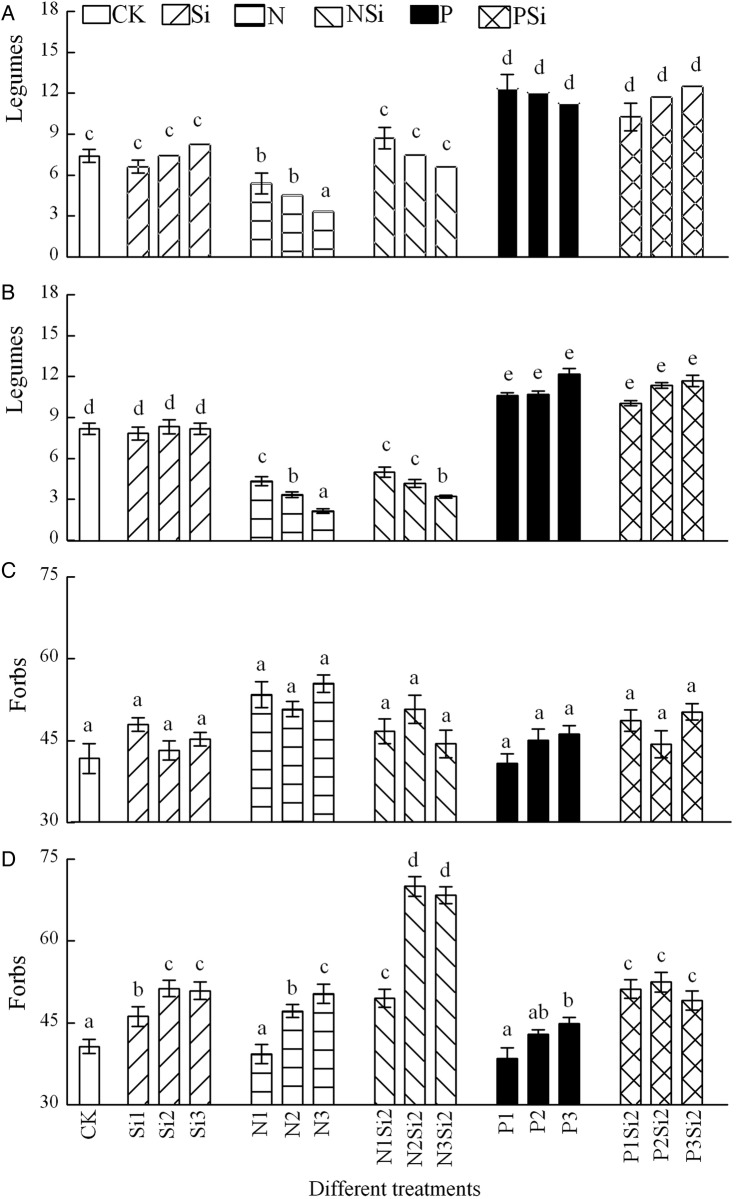
Aboveground primary productivity in groups of legumes (A and B) and forbs (C and D) after Si, N and P addition in 2012 (A and C) and 2013 (B and D), *n* = 6. Different letters above bars indicate significant difference between different treatments.

Silicon and P addition alone or Si addition with N or P included had no effect on APP of grasses and sedges in 2012 (Fig. 3A and C). Aboveground primary productivity of grasses (*F* = 53.674, df = 5, *P* < 0.001) (Fig. 3B) and sedges (*F* = 11.024, df = 5, *P* < 0.001) (Fig. 3D) was significantly higher in NSi treatments than N or Si treatments in 2013. Aboveground primary productivity of legumes decreased with the levels and amounts of N addition (*F* = 72.514, df = 3, *P* < 0.001) and increased with P addition (*F* = 47, df = 3, *P* < 0.001) (Fig. 4A and B). Different fertilization combinations did not affect the APP of forbs in 2012 (Fig. 4C). Silicon in combination with N resulted in higher APP of forbs than N (*F* = 48.102, df = 5, *P* < 0.001) or P (*F* = 19.452, df = 5, *P* < 0.001) addition alone in 2013 (Fig. 4D). Two-way ANOVA revealed that there were significant interaction effects between Si and N in APP of forbs (*F* = 51.88, df = 4, *P* = 0.035) in 2013 (Fig. 4D), and between different years in all functional groups (Table 2).

### Species richness of four functional groups

**Figure 5.**
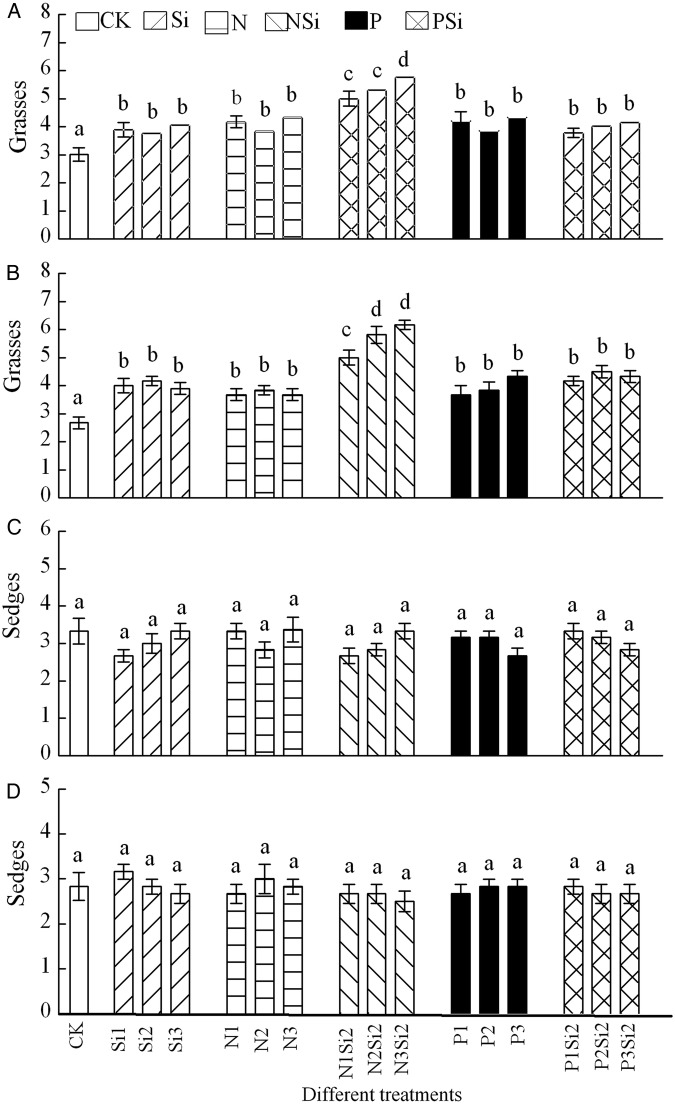
Species richness in groups of grasses (A and B) and sedges (C and D) after Si, N and P addition in 2012 (A and C) and 2013 (B and D), *n* = 6. Different letters above bars indicate significant difference between different treatments.

**Figure 6.**
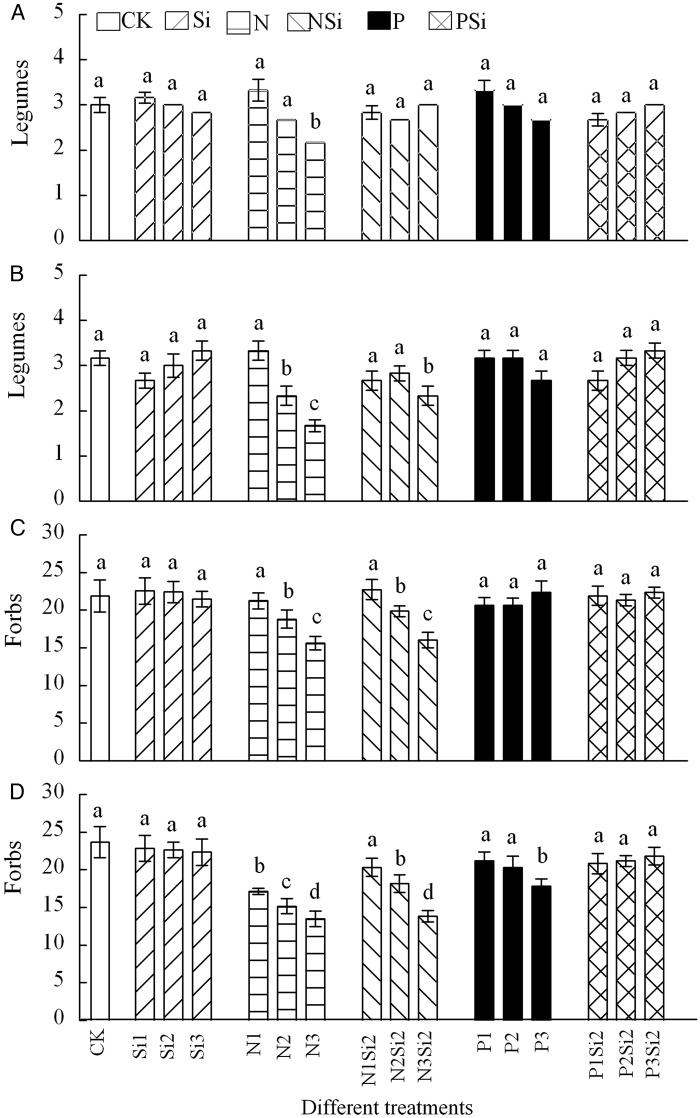
Species richness in functional types of legumes (A and B) and forbs (C and D) after Si, N and P addition in 2012 (A and C) and 2013 (B and D), *n* = 6. Different letters above bars indicate significant difference between different treatments.

Nitrogen and Si addition together led to significantly higher species richness of grasses than N or Si addition alone (*F* = 22.047, df = 5, *P* < 0.001) (Fig. 5A and B). There were no significant differences of species richness of sedges among the different treatments in 2012 (Fig. 5C) and 2013 (Fig. 5D). Species richness of legumes decreased with the increasing levels and amounts of N addition in 2012 (*F* = 7.027, df = 3, *P* < 0.018) and 2013 (*F* = 12.071, df = 3, *P* < 0.001) (Fig. 6A and B). Species richness of forbs decreased with the increasing amounts of N and NSi addition (Fig. 6C and D). Adding Si with N or P resulted in higher species richness of forbs than addition of N (*F* = 45.615, df = 5, *P* < 0.001) or P (*F* = 8.598, df = 5, *P* < 0.001) alone in 2013. A highly significant interaction between N and Si (*F* = 44.12, df = 4, *P* = 0.047) was found in species richness of forbs in 2013, and between different years in forbs and legumes groups (Table 2).

## Discussion

### Effects of Si, N and P addition on APP

Our present study showed that Si addition alone significantly increased the APP of the whole community and the functional groups of grasses, sedges and forbs in an alpine meadow in 2013. Some researchers have reported that Si elevated biomass, crop yield and photosynthesis and postponed leaf senescence (Cooke and Leishman 2011; Detmann *et al*. 2012; Farooq *et al*. 2013). The different responses of the four functional groups may be related to the different accumulation of Si in plants. Some results reported that Si accumulation has been found to a greater extent. Plants of the families Poaceae, Equisetaceae and Cyperaceae show high Si accumulation (0.4 % Si) and the Cucurbitales, Urticales and Commelinaceae show intermediate Si accumulation (2–4 % Si), while most other species demonstrate little accumulation (Hodson *et al*. 2005; Currie and Perry 2007).

The present study showed that a highly significant interaction between Si and N was found on APP and species richness of the whole community and the functional group of forbs in an alpine meadow. This beneficial effect of Si has been traditionally attributed to an increase in the erectness of leaves, allowing better light transmittance through plant canopies and thus indirectly improving whole-community photosynthesis (Tamai and Ma 2008), especially for the shorter species of forbs.

Findings from our study corroborate and expand on the results of Ma *et al*. (2014) and Ren *et al*. (2010) who documented the response of aboveground vegetation during short-term fertilization addition. Our study provides compelling evidence that N rather than P is the primary limiting nutrient for vegetation. Our results are similar to those of Ren *et al*. (2010) and Onipchenko *et al*. (2012) in that N additions continued to promote significantly greater growth response than P additions or control conditions.

Our result regarding the four functional groups is in line with studies in alpine grassland (Luo *et al*. 2006; Niu *et al*. 2014). The different responses of the four groups to treatment with different resources have been explored by many researchers (Gough *et al*. 1994; Rajaniemi 2003; Herbert *et al*. 2004). Legumes, due to their N-fixing ability, often respond positively to P and negatively to N fertilization in alpine plant communities (Bowman *et al*. 1993; Theodose and Bowman 1997). Our results support these observations. Graminoids (grasses and sedges) usually increase their abundance after N treatment in alpine communities (Calvo *et al*. 2005). Grasses and sedges can respond differently to N fertilization. Our results demonstrate that grasses respond to N fertilization better than sedges, which is not in line with a study by Onipchenko *et al*. (2012) who found that sedges respond to N fertilization better than grasses in the Alps. This may be due to the low amount of N and P applied. Positive responses of forbs to fertilization were noted in several studies (Madaminov and Budtueva 1990; Calvo *et al*. 2005).

### Effects of Si, N and P addition on plant species richness

Reduced species richness following N or P supply in an alpine meadow, as observed in this study, is consistent with the widely demonstrated declines in species richness with N or P enrichment occurring in various terrestrial ecosystems (Stevens *et al*. 2004; Suding *et al*. 2005). Several mechanisms have been used to account for the reduction in species diversity under elevated N or P (Gough *et al*. 1994; Rajaniemi 2003; Suding *et al*. 2005). For example, Hautier *et al*. (2009) and Rajaniemi (2003) showed that the supply of resources may enhance APP and then lead to decreased plant diversity. At high APP, competition between species shifted from belowground competition for soil resources to aboveground competition for light. Under this condition, plant species became light limited, which led to faster-growing or taller species displacing inferior species by size-asymmetric competition for light (Pennings *et al*. 2005; Vojtech *et al*. 2007). Thus, differences in height and differential responses of height to light competition among species are likely to be major causes of plant species loss in a plant community following addition of limiting resources.

For the four functional groups, species richness differed in their response to different fertilization. The positive response of grasses could have been primarily ascribed to the enhancement of dominant species owing to their ability to quickly explore available resources relative to other species (Yuan *et al*. 2005). On the contrary, the species of sedges and forbs are slower-growing or shorter, so they had the disadvantage of having to compete for light more than grasses. The competitive advantage of legume species is proposed to decline with increased N availability because of their inherent N-fixing characteristics (Xia and Wan 2008).

## Conclusions

In summary, our experimental results demonstrated that (i) Si, N or P addition increased APP, and the highest APP occurred when the highest level of N was added, which showed that N rather than P is the primary limiting nutrient for vegetation in this region; (ii) N or P addition decreased the community species richness, but Si, N or P addition increased the species richness of grasses and (iii) N addition with Si significantly increased APP, but also significantly alleviated the loss of species richness caused by N addition, especially for the group of forbs. Our findings will together help to improve our ability to predict community composition and biomass dynamics in alpine meadow community subject to external nutrient inputs (Bobbink *et al*. 2010).

## Sources of Funding

This study was financially supported by the open project program of State Key Laboratory of Grassland Agro-ecosystems and the National Natural Sciences Foundation of China (no. 41430749, 31370423 and 41171046). This work was done in the Research Station of Alpine Meadow and Wetland Ecosystems of Lanzhou University.

## Contributions by the Authors

D.X. and G.D. designed the research; D.X. and T.G. analysed the data and led the writing. X.F., H.B. and R.Z. conceived the experiments and oversaw the collection of field and laboratory data.

## Conflict of Interest Statement

None declared.
